# Genistein Blunted Detrimental Effects of Polycystic Ovary Syndrome on the Ovarian Tissue of Rats by Improving Follicular Development and Gonadotropin Secretion

**DOI:** 10.5935/1518-0557.20210089

**Published:** 2022

**Authors:** Samran Khezri, Alireza Alihemmati, Ali Abedelahi

**Affiliations:** 1 Stem Cell Research Center, Tabriz University of Medical Sciences, Tabriz, Iran; 2 Department of Anatomical Sciences, Faculty of Medicine, Tabriz University of Medical Sciences, Tabriz, Iran; 3 Department of Reproductive Biology, Faculty of Advanced Medical Sciences, Tabriz University of Medical Sciences, Tabriz, Iran

**Keywords:** genistein, polycystic, ovarian tissue, follicular competence, gonadotropins

## Abstract

**Objective:**

Polycystic ovary syndrome (PCOS) is a common cause of female infertility worldwide. It has been shown that genistein, a natural isoflavone, may influence follicular competence via the production of gonadotropins in women with PCOS. The current study aims to evaluate the effects of genistein on the ovarian tissue of rats with PCOS.

**Methods:**

Thirty female Wistar rats were randomly divided into the following four groups: Control; PCOS (rats received 2 mg/kbW estradiol valerate); Genistein (rats given 1 mg/kg BW of genistein for 14 days); and Genistein + PCOS. All animals were slaughtered under anesthesia and blood samples were collected for biochemical analysis. Follicular morphology was analyzed based on histologic examination.

**Results:**

Histologic examination exhibited enhanced follicular atresia at various stages in the rats with PCOS compared to controls (*p*<0.001). Induction of PCOS caused significant reduction in gonadotropin levels and steroid hormone levels consistent with insulin resistance (*p*<0.01). Data showed that 14-day administration of genistein might improve follicular morphology in rats with PCOS (*p*<0.001). Genistein treatment increased the production of gonadotropins and steroid hormones and alleviated insulin resistance in Rats with PCOS (*p*<0.001).

**Conclusions:**

This study indicated that genistein treatment exerted a beneficial effect on the ovarian tissue of rats with PCOS by improving follicular growth and hormone balance.

## INTRODUCTION

Polycystic ovary syndrome (PCOS) affects 5-10% of women of reproductive age and is the most common endocrine disorder of this population (Azziz *et al*., 2004; Azziz, 2018). The main symptoms of PCOS are related to the elevation of androgens, menstrual disorders, and anovulatory infertility. This syndrome can cause deleterious effects on the general physiology and metabolic rates. For example, individuals with PCOS are at greater risk of developing insulin resistance, hypertension, abdominal obesity, high blood pressure, type 2 diabetes, and cardiovascular disease (Behboodi Moghadam *et al*., 2018). It has been proposed that the hypothalamic-pituitary-gonadal (HPG) axis is responsible for functional alterations and development of PCOS (Ferreira & Motta, 2018). In progressive PCOS conditions, the serum levels of luteinizing hormone (LH) and follicle-stimulating hormone (FSH) are increased and reduced, respectively, leading to elevated LH/FSH ratios and excessive ovarian androgen synthesis (Marx & Mehta, 2003; Pasquali *et al*., 2016).

In recent decades, natural phytochemicals such as phytoestrogens have been widely used in the treatment of patients with PCOS (Eslamian & Hekmatdoost, 2019). Phytoestrogens are natural functional estrogen-like compounds that can be found in plant foods (Moutsatsou, 2007). Among these compounds, genistein is a bioactive isoflavone that originates from precursors such as biochanin A and formononetin (Theil *et al*., 2011). Genistein plays an important role in the management and treatment of many diseases including diabetes, menopausal symptoms, osteoporosis, cardiovascular and kidney disease (Setchell, 2006). There has been growing interest in the biological properties of this compound in traditional medicine. Similar to 17β-estradiol, genistein binds to uterine and ovarian estrogen receptors (ER) (Pelletier & El-Alfy, 2000; Nynca *et al*., 2015). A plethora of experiments has confirmed the efficiency of genistein in PCOS patients. However, the underlying mechanisms of genistein in the mediation of steroidogenic and gonadotropic activity are poorly understood in PCOS. Moreover, there is still little data on the possible role of genistein in insulin resistance secondary to PCOS. In this regard, the present study aims to determine the beneficial effects of genistein on follicular structure, sex hormone profile, and insulin resistance in a rat model of PCOS.

## MATERIALS AND METHODS

### Animal issues and Experimental Design

All chemicals were purchased from Sigma-Aldrich unless stated otherwise. In this study, 30 female Wistar albino rats (aged 8 weeks and weighing 200±20 g on average) were prepared and maintained under standard conditions (12-hour light/ 12-hour dark cycles) at a constant temperature of 21±2°C, with free access to chewing food and water. All steps of this study were approved by the Local Ethics Committee of Tabriz University of Medical Sciences (IR.TBZMED.VCR.REC.1394.2-5/6). Rats with regular sexual cycles were randomly divided into the following four groups (n=7/each): Control; PCOS; Genistein; and Genistein plus PCOS.

### Evaluation of the estrous cycle

The sexual cycle status of the rats was carefully evaluated daily at 10:00 AM by vaginal smear analysis one month before induction of PCOS (Sun *et al*., 2013). The smears were viewed on a light microscope (Olympus, Japan) at high magnification (400X) and the existence of all cell types such as infiltrated leukocytes, nucleated and cornified epithelial cells was monitored.

### Induction of PCOS in a rat model

Estradiol valerate (2 mg/kg body weight (BW); Aburaihan Co., Iran) was subcutaneously injected for 60 consecutive days (Shi & Vine, 2012). In the rats with PCOS, irregular cycles were indicated with prominent vaginal cornification in vaginal smears. Besides, the efficiency of PCOS induction was further confirmed by monitoring ovarian histology and serum gonadotropin levels.

### Preparation of Genistein

About 0.2 mg genistein (Santa Cruz Co, USA) was dissolved in dimethyl sulfoxide (DMSO) under standard conditions - away from sunlight, moisture, microbial contamination - and then stored at -20°C until use. In the PCOS + Genistein and Genistein groups, 1 mg/kbW of genistein was subcutaneously injected for 14 days after induction of PCOS.

### Evaluation of ovarian histology and follicle count

After the completion of the experimental protocol, 20 rats were randomly selected and slaughtered under deep anesthesia. The ovaries were harvested and surrounding adipose tissues were removed. Tissues were fixed in 10% formaldehyde solution, dehydrated in increasing alcohol concentrations, and clarified using xylene. The specimens were embedded in paraffin blocks, and 5-µm thick serial sections were prepared. The procedure was continued with deparaffinization in xylene and staining of the slides with hematoxylin and eosin (H&E). Tissues were observed under a light microscope at 400x magnification. The number of follicles at various stages of development and corpus lutea were counted in serial sections. The thicknesses of theca and granulosa layers of the antral follicles were carefully assessed. In this study, follicle classification was done according to the size and shape of the follicles and the granular cell layer at the periphery of oocytes (Ghavami *et al*., 2015). Follicles were classified into primary follicles (single layer of cuboidal granulosa cells around oocytes), preantral follicles (multilayer of granulosa cells around oocytes with no antral cavity), and antral follicles (indicated with numerous granulosa cells with antral cavity and cumulus granulosa cell layer). Follicular quality was scored as normal (intact oocyte and granulosa cells); atretic follicles (abnormal structure with pycnotic and irregularly shaped granulosa cells and oocytes) (Brawer *et al*., 1986).

### Ovarian follicular viability

**Figure 1:**
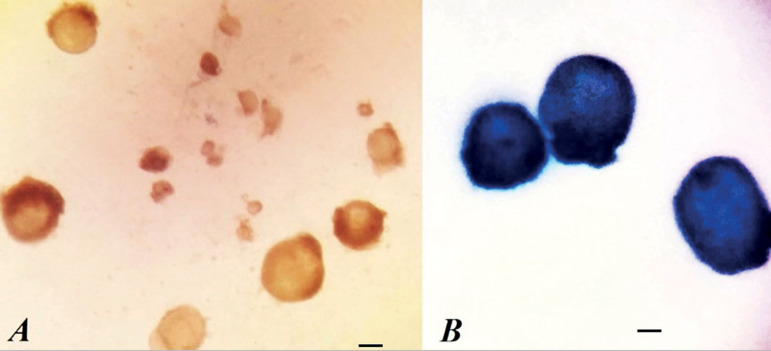
Viable ovarian follicles (A) and degenerated follicles (B) after trypan blue staining Scale bar= 100µm.

Follicles at different stages of development were mechanically isolated using 29-gauge needles under a stereomicroscope (SZ-STS, Olympus, Tokyo, Japan) and were transferred into 20 µl microdroplets covered with mineral oil. The isolated follicles were selected according to the central oocyte with membrane-enclosed granulosa cells (Fig. 1A-B). Follicles were stained with 0.4% trypan blue and visualized using an inverted microscope (Olympus, Japan). The follicles were scored as viable (if the oocyte and surrounding granulosa cells were not stained) or degenerated follicles (if they stained blue) (Sanfilippo *et al*., 2011).

### Measuring gonadotropins and steroidal hormones

After the last treatment, blood samples were collected and centrifuged at 3000 g for 10min at 4˚C. The serums were separated and stored at -80°C until hormonal analysis. Concentrations of serum gonadotropin (FSH and LH) and steroidal hormones (estradiol, progesterone, and testosterone) were measured using a commercial Enzyme-Linked Immune Sorbent Assay (ELISA) kits according to the manufacturer's instructions (Monobind, Inc., Lake Forest, USA). All samples were assayed in duplicate.

### Insulin resistance assay

Blood samples were collected from the tail vein and the levels of fasting blood glucose and fasting insulin were measured using Homeostasis Model Assessment - insulin resistance (HOMA-IR). Serum levels of lipids and glucose were calculated using a Siemens Dimension MAX system (Siemens Healthcare Diagnostics Inc.). Plasma insulin was evaluated by magnetic affinity immunoassay (Insulin MPAIA Kit). HOMA-IR was calculated according to the methods described by Matthews *et al.* (1985).

### Statistical analysis

Statistical analysis was performed using SPSS (ver. 20). Data were expressed as mean ± standard deviation (SD). Statistical differences between groups were analyzed by one-way analysis of variance (ANOVA). A probability level of *p*<0.05 was considered to be statistically significant.

## RESULTS

### Genistein reduced the detrimental effect of PCOS on ovarian tissue

**Figure 2:**
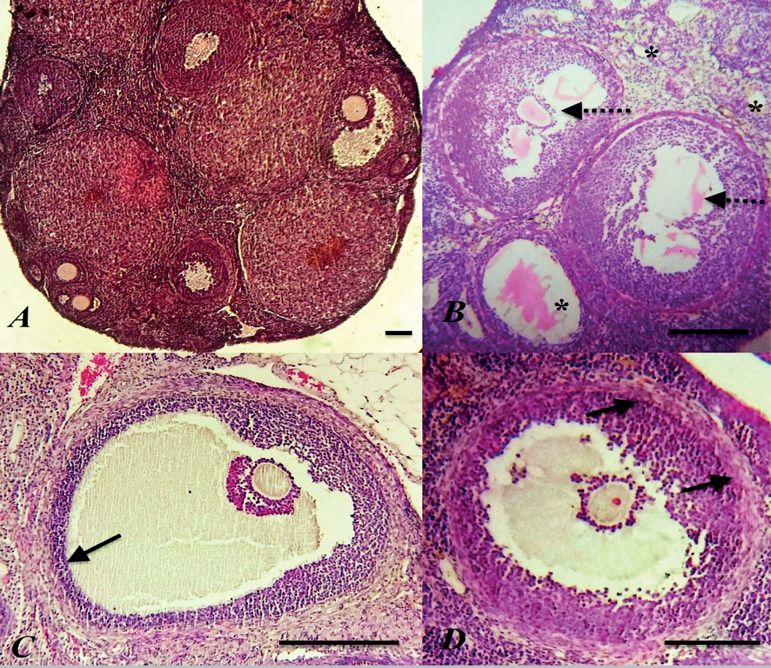
Histomorphology of ovarian follicles. (A), control group (B), atretic follicles (star), degenerated oocytes, and granulosa cells (dash arrow) in the PCOS group (C), the granulosa cells layer decreased and (D), theca cells layer increased in the PCOS group (black arrow) Scale bar= 100µm.

**Figure 3:**
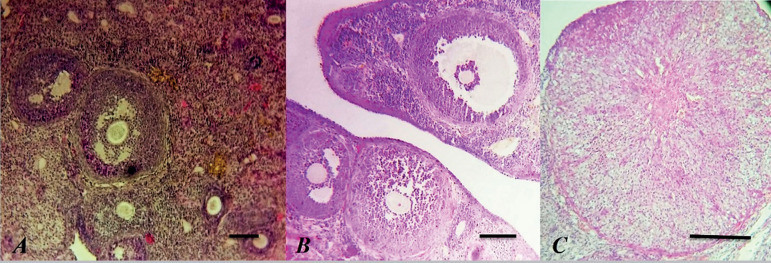
Histomorphology of ovarian follicles. (A), genistein treated in the non-PCOS group (B), genistein treated in PCOS group, and (C), corpora lutea (CL) in genistein treated group GC: granulosa cell layer; TL: theca cell layer, Scale bar= 100µm.

Histologic examination revealed normal follicular morphology at different stages of development (primary, preantral, and antral follicles) in the control rats. Data showed that the corpus luteum and interstitial tissue were histologically normal (Fig. 2A). In contrast, atretic changes were evident in ovarian follicles in different stages of development after the induction of PCOS (Fig. 2B). In the rats with PCOS, remarkable degeneration was indicated with a thin granulosa layer and abnormally thickened theca layer in some large follicles (Fig. 2C-D). These features coincided with the lack of corpora lutea in histology slides, showing the inhibition of ovulation in rats with PCOS. In the genistein-treated rats, follicles had normal granulosa cell layers and noticeable theca layers (Fig. 3A-B) and corpora lutea (Fig. 3C). These data demonstrate that the 14-day administration of genistein can restore ovarian tissue function in rats with PCOS.

### Histomorphometric evaluation of ovarian follicles

Table 1 shows the histologic characteristics of follicles at various stages of development. A total of 542 primary follicles (135 per group) were histologically examined in all groups. Rats with PCOS had a significantly higher percentage of primary atretic follicles than controls (13 *vs*. 2%; *p*<0.001). According to our data, genistein-treated rats had a higher proportion of healthy follicles than rats with PCOS (98% *vs*. 87%; *p*<0.001). Normality of preantral follicles was evaluated based on the examination of 424 preantral follicles (106 per group). Results revealed that the percentage of normal preantral follicles was significantly reduced in rats with PCOS as compared to controls (86% *vs*. 98%; *p*<0.01). Consistently, the number of atretic follicles increased following the onset of PCOS (14% *vs*. 2%; *p*<0.01). Genistein had the potential to increase the number of normal preantral follicles after administration to rats with PCOS (94% *vs*. 86%; *p*<0.01). More specifically, the percentage of atretic preantral follicles was significantly decreased (6% *vs*. 14%; *p*<0.01) in comparison with rats with PCOS. The assessment of normality of antral follicles was based on the findings from 370 antral follicles (92 per group). Interestingly, the number of atretic antral follicles increased significantly in rats with PCOS after normal rats received genistein for 14 days in comparison with controls (19% *vs*. 5%; *p*<0.001). The treatment of rats with PCOS with genistein increased the percentage of normal antral follicles compared untreated rats with PCOS (95% *vs*. 81%; *p*<0.001).

**Table 1.** Number and percentage of intact (int) and atretic (atr) follicles in various stages of development after Hematoxylin-Eosin staining.

Group	Primary follicles (%)			Preantral follicle (%)			Antral follicle (%)		
	total	int	atr	total	int	atr	total	int	Atr
Control	152	150 (98)	2 (2)	114	112 (98)	2 (2)	109	107 (98)	2 (2)
Gen	140	137 (97)	3 (3)	110	103 (94)	7 (6)	98	93 (95)	5 (5)
PCOS	112	98 (87)^a^ †	14 (13)^a^†	92	79 (86)^a^*	13 (14)^a^*	75	61 (81)^a^†	14 (19)^a^†
Gen+PCOS	138	136 (98)	2 (2)	108	102 (94)	6 (6)	88	84 (95)	4 (5)

a Represents a significant difference between PCOS and other groups.

* *p*<0.01,

† *p*<0.001.

Gen: genistein; PCOS: polycystic ovary syndrome

### Number of Corpus Luteum and Granulosa Cells and Theca Layer Thickness

In rats with PCOS, absence of the corpus luteum and thinning of the granulosa layer were associated with PCOS. According to our data, the thickness of the theca layer was significantly increased in the Genistein group as compared to controls (Table 2, *p*<0.001). Likewise, administration of genistein to rats with PCOS for 14 days increased the number of corpora lutea and improved the thickness of granulosa cells. As expected, theca layer thickness decreased in rats with PCOS given genistein (*p*<0.001). Interestingly, granulosa and theca layer thickness increased in the genistein group compared to controls (Table 2, *p*<0.01).

**Table 2.** Comparative assessment (mean ± SD) of the thickness of the granulosa layer (mm), thickness of the theca layer (mm) in the antral follicles, and number of corpora lutea in the ovaries of control and treated groups.

Groups	Granulosa layer	Theca layer	Corpora lutea
Control	33.40±1.14^b^*	24.60±1.14^b^*	2.8±0.44
Gen	29.80±0.83	21.40±1.51	2.4±0.54
PCOS	19.60±0.80^a^†	33.00±1.22^a^†	0.0±0.00^a^†
Gen+PCOS	29.80±1.48	21.00±1.23	2.2±0.44

a Represents a significant difference between PCOS and other groups.

b Represents a significant difference between control and other groups.

* *p*<0.01,

† *p*<0.001.

Gen: genistein; PCOS: polycystic ovary syndrome;

### Follicle Viability

The percentages of viable follicles isolated from different groups were examined after trypan blue staining (Table 3). Data revealed that the viability of primary follicles (*p*<0.001), preantral follicles, and antral follicles (*p*<0.01) was decreased in rats with PCOS and that administration of genistein might restore the viability of follicles. These results were consistent with the normality of follicles at various stages of development.

**Table 3.** Number and percentage of intact (int) and degenerated (deg) follicles in various stages of development after trypan blue staining.

Group	Primary follicles (%)			Preantral follicle (%)			Antral follicle (%)		
	total	int	deg	Total	int	deg	total	int	deg
Control	74	72 (98)	2 (2)	64	62 (98)	2 (2)	71	68 (96)	3 (4)
Gen	71	69 (98)	2 (2)	61	58 (96)	3 (4)	68	66 (98)	2 (2)
PCOS	45	37 (83)^a^†	8 (17)^a^†	49	41 (83)^a^*	8 (17)^a^*	46	39 (84)^a^*	7 (6)^a^*
Gen+PCOS	70	68 (98)	2 (2)	58	55 (96)	3(4)	70	68 (98)	2 (2)

a Represents a significant difference between PCOS and other groups.

* *p*<0.01,

† *p*<0.001.

Gen: genistein; PCOS: polycystic ovary syndrome.

### Gonadotropin and steroidal hormones

The serum levels of gonadotropins and steroidal hormones were measured in all groups (Table 4). Rats that received estradiol valerate exhibited a significant increase in LH levels along with a reduction in FSH levels in comparison with controls (*p*<0.01). Genistein treatment decreased the abnormal elevation of LH levels (*p*<0.01) and increased FSH (*p*<0.001) to near-control levels in rats with PCOS in comparison to all groups. We found a noticeable elevation in testosterone and estradiol levels (*p*<0.001) and a reduction in progesterone levels (*p*<0.01) in rats with PCOS in comparison with controls. Following genistein administration, serum levels of sex-related hormones got to near-normal levels.

**Table 4.** Comparative assessment (mean ± SD) of serum concentrations of sex steroids in control and experimental groups

	Control	Gen	PCOS	Gen+PCOS
FSH (IU/L)	0.47±0.04	0.43±0.02	0.22±0.02^a^†	0.40±0.02
LH (IU/L)	0.45±0.05	0.42±0.02	0.57±0.03^a^*	0.40±0.01
Estradiol (ng/ml)	1.19±0.01	1.18±0.00	1.24±0.01^a^†	1.19±0.00
Progesterone (ng/ml)	11.47±0.45	11.16±0.04	10.07±0.07^a^*	11.08±0.10
Testosterone (ng/ml)	4.42±0.01	4.35±0.02	5.05±0.05^a^†	4.34±0.02
Glucose (mM/L)	11.23±0.03	10.89±0.04	18.13±0.08^a^†	14.03±0.02
Insulin (MU/L)	38.42±0.10	39.03±0.08	61.12±0.15^a^†	40.12±0.09
HOMA-IR	19.17±0.06	20.01±0.08	49.24±0.12^a^†	25.01±0.10

a Represents a significant difference between PCOS and other groups.

* *p*<0.01,

† *p*<0.001.

Gen: genistein; PCOS: polycystic ovary syndrome; FSH: follicle-stimulating hormone; LH: Luteinizing hormone; HOMA-IR: Homeostasis Model Assessment- Insulin Resistance.

### Glucose, insulin levels, and insulin resistance

Our data showed that insulin and glucose levels were increased in rats with PCOS. Insulin resistance was also detected in rats after estrogen injections were administered for 60 days in comparison with controls (Table 4, *p*<0.001). We noted that genistein reversed these conditions and returned parameters to near-control levels. Following genistein administration, a significant decrease in insulin and glucose levels and insulin resistance were observed in rats with PCOS (*p*<0.001). Data showed that genistein reduced abnormal metabolic hormone levels in rats with PCOS.

## DISCUSSION

This study tried to show the protective effects of genistein on ovarian function in a rat model of PCOS. To this end, different parameters such as ovarian morphology, gonadotropin levels, steroidogenesis, and insulin resistance were examined pre- and post-genistein administration in rats with PCOS. Our data confirmed the appearance of numerous atretic follicles as a common feature of ovarian insufficiency in rats with PCOS. Pathological changes such as multi-sized cystic follicles with a thin layer of granulosa cells and hypertrophic theca layers were observed. The connection between oocytes and surrounding granulosa cells was also disrupted. Absence of corpora lutea and anovulation were also observed.

Consistent with our data, Rajaei *et al*. (2019) found numerous cystic follicles with degenerated granulosa and thickened theca layers in rats with PCOS. It has been suggested that prolonged estradiol administration might induce follicular atresia and alter resting follicles. In line with these descriptions, it is noteworthy to mention that abnormal folliculogenesis is the most important complication in PCOS.

We also found that genistein preserved follicular morphology at different stages of development in rats with PCOS, which thus allows growing follicles to develop into the antral phase and mature (Zhuang *et al*., 2010; Medigović *et al*., 2012). Estrous cycle determination is a useful method for monitoring the reservation of follicles. Both histologic and biochemical tests showed that rats with PCOS had irregular estrous cycles. These results may be related to changes in the levels of gonadotropins and steroid hormones (Azziz, 2006; Paixão *et al*., 2017). These hormones have a pivotal role in the regulation of the estrous cycle and in follicular maturation (Sun *et al*., 2013). Similarly, Badawy & Elnashar (2011) indicated that imbalances in the levels of FSH and LH might lead to irregular estrous cycles in subjects with PCOS.

The rats with PCOS given genistein returned gradually to having normal estrous cycles; follicular development improved and follicles were spared from PCOS-related injuries. It has been postulated that genistein might directly affect the HPG axis and elevate gonadotropin-releasing hormone (GnRH) levels. The regulation of the HPG axis can support the production of gonadotropin hormones including FSH and LH (Flynn *et al*., 2000; Ren *et al*., 2001; Zin *et al*., 2013; Ali *et al*., 2020). FSH decreases in rats with PCOS impairs follicular growth and maturation, leading to the formation of multiple cysts inside the ovarian tissue parenchyma. Along with these changes, excessive LH production induces the synthesis of testosterone in ovarian tissue and promotes irregular estrous cycles and abnormal ovulation (Tena *et al*., 2011). In terms of gonadotropin synthesis and steroidogenesis, synergic coordination of granulosa and theca cells is vital for the normal physiology of growing follicles (Kafali *et al*., 2004).

In agreement with the results published by Kafali *et al*. (2004), our data suggested that the multiple cysts seen in PCOS might be associated with poor interaction between granulosa and theca cells following imbalances in FSH and LH levels. Therefore, folliculogenesis is impaired in rats with PCOS due to decreases in the number of granulosa cells, abnormal theca layer thickening, and androgen production. Our findings also suggest that high levels of estradiol in rats with PCOS impair ovarian function and increase the number of atretic follicles at various developmental stages. Increased estradiol exerts a positive feedback on the HPG axis, leading to disruption of follicle growth and anovulation (Schoeters *et al*., 2008). Schoeters *et al*. (2008) showed that the administration of genistein sets testosterone and estradiol levels back to normal.

Likewise, the elevation of FSH in rats with PCOS stimulates the production of estrogen from the follicles and improves ovarian follicles by decreasing atretic changes. Considering the therapeutic effects of genistein, it is noteworthy to mention that genistein, as a phytoestrogen, has a similar structure to 17β-estradiol. The anti-estrogenic activity of genistein is related to its affinity to estrogen receptors located primarily in the uterus and ovarian tissue (Pelletier & El-Alfy, 2000). The reduction of testosterone in rats with PCOS after treatment with genistein was possibly caused by the inhibition of aromatase activity (conversion of androgen hormones into estrogens) induced by estradiol components (Weber *et al*., 2001; Rajan *et al*., 2017). It has been postulated that increases in testosterone might postpone follicular maturation and arrest follicular ovulation, as commonly seen in rats with PCOS (Henmi *et al*., 2001; Beloosesky *et al*., 2004). To date, the exact effect of genistein on androgen production is unclear. However, it has been proposed that genistein might decrease testosterone levels in rats with PCOS by interfering with steroidogenesis in the adrenal gland via reduction of intracellular cAMP and promotion of aromatase activity (Ohno *et al*., 2003; Myllymäki *et al*., 2005). It should be noted that theca cells are responsible for providing the androgen substrate for aromatase activity in granulosa cells (Magoffin & Weitsman, 1993).

Our data confirmed that a thickened theca layer in the follicles might inhibit aromatase activity, leading to increased androgen levels in rats with PCOS. Progesterone is a necessary indicator for ovulation (Skrtic *et al*., 2011). This study suggested that progesterone levels were significantly decreased in rats with PCOS due to the reduction of ovarian corpus luteum. Genistein treatment increased progesterone levels in rats with PCOS, thereby allowing follicle maturation and ovulation. Insulin resistance and hyperinsulinemia may impair gonadotropin secretion in women with PCOS and support LH elevation (Tosi *et al*., 2012). In line with this statement, hyperinsulinemia appears to be an important factor in maintaining hyperandrogenemia, thereby increasing the synthesis of androgens in theca cells and granulosa cells. Therefore, these alterations arrest follicular maturation and result in disruption of ovulation in rats with PCOS (Barber *et al*., 2016; Dupont & Scaramuzzi, 2016). Another reason would be that insulin inhibits the synthesis of sex hormone-binding globulin, and thus increases the concentration of serum testosterone (Pasquali *et al*., 1995). Previous studies proved the therapeutic effects of genistein on diabetic animal models and postmenopausal women via the regulation of insulin and glucose metabolism (Guo *et al*., 2014; El-Kordy & Alshahrani, 2015; Liu *et al*., 2017). Similarly, genistein treatment decreased insulin markers in rats with PCOS such as insulin and glucose, as well as insulin resistance. Therefore, follicular viability and ovarian function improved by regulating insulin resistance and sensitivity.

## CONCLUSION

Genistein exerts beneficial effects on rats with PCOS by regulating gonadotropin and androgen synthesis, controlling insulin resistance, and allowing prompt follicular development and function.

## References

[r1] Ali M, Broyles TM, Davis LK, Gonzalez CMF, Lucero D, Stary L, Guarraci FA (2020). Neonatal exposure to genistein affects reproductive physiology and behavior in female and male Long-Evans rats. Behav Pharmacol.

[r2] Azziz R, Woods KS, Reyna R Key TJ, Knochenhauer ES, Yildiz BO (2004). The prevalence and features of the polycystic ovary syndrome in an unselected population. J Clin Endocrinol Metab.

[r3] Azziz R, Nestler J, Dewailly D (2006). Androgen excess disorders in women: polycystic ovary syndrome and other disorders.

[r4] Azziz R (2018). Polycystic ovarian syndrome in the ovary. Obstet Gynecol.

[r5] Badawy A, Elnashar A (2011). Treatment options for polycystic ovary syndrome. Int J Womens Health.

[r6] Barber TM, Dimitriadis GK, Andreou A, Franks S (2016). Polycystic ovary syndrome: insight into pathogenesis and a common association with insulin resistance. Clin Med (Lond).

[r7] Behboodi Moghadam Z, Fereidooni B, Saffari M, Montazeri A (2018). Measures of health-related quality of life in PCOS women: a systematic review. Int J Womens Health.

[r8] Beloosesky R, Gold R, Almog B, Sasson R, Dantes A, Land-Bracha A, Hirsh L, Itskovitz-Eldor J, Lessing JB, Homburg R, Amsterdam A (2004). Induction of polycystic ovary by testosterone in immature female rats: Modulation of apoptosis and attenuation of glucose/insulin ratio. Int J Mol Med.

[r9] Brawer JR, Munoz M, Farookhi R (1986). Development of the polycystic ovarian condition (PCO) in the estradiol valerate-treated rat. Biol Reprod.

[r10] Dupont J, Scaramuzzi RJ (2016). Insulin signalling and glucose transport in the ovary and ovarian function during the ovarian cycle. Biochem J.

[r11] El-Kordy EA, Alshahrani AM (2015). Effect of genistein, a natural soy isoflavone, on pancreatic β-cells of streptozotocin-induced diabetic rats: Histological and immunohistochemical study. J Microsc Ultrastruct.

[r12] Eslamian G, Hekmatdoost A (2019). Nutrient Patterns and Risk of Polycystic Ovary Syndrome. J Reprod Infertil.

[r13] Ferreira SR, Motta AB (2018). Uterine Function: From Normal to Polycystic Ovarian Syndrome Alterations. Curr Med Chem.

[r14] Flynn KM, Ferguson SA, Delclos KB, Newbold RR (2000). Effects of genistein exposure on sexually dimorphic behaviors in rats. Toxicol Sci.

[r15] Ghavami M, Mohammadnejad D, Beheshti R, Solmani-Rad J, Abedelahi A (2015). Ultrastructural and Morphalogical Changes of Mouse Ovarian Tissues Following Direct Cover Vitrification with Different Cryoprotectants. J Reprod Infertil.

[r16] Guo TL, Wang Y, Xiong T, Ling X, Zheng J (2014). Genistein modulation of streptozotocin diabetes in male B6C3F1 mice can be induced by diet. Toxicol Appl Pharmacol.

[r17] Henmi H, Endo T, Nagasawa K, Hayashi T, Chida M, Akutagawa N, Iwasaki M, Kitajima Y, Kiya T, Nishikawa A, Manase K, Kudo R (2001). Lysyl oxidase and MMP-2 expression in dehydroepiandrosterone-induced polycystic ovary in rats. Biol Reprod.

[r18] Kafali H, Iriadam M, Ozardalı I, Demir N (2004). Letrozole-induced polycystic ovaries in the rat: a new model for cystic ovarian disease. Arch Med Res.

[r19] Liu Y, Li J, Wang T, Wang Y, Zhao L, Fang Y (2017). The effect of genistein on glucose control and insulin sensitivity in postmenopausal women: A meta-analysis. Maturitas.

[r20] Magoffin DA, Weitsman SR (1993). Differentiation of ovarian theca-interstitial cells in vitro: regulation of 17 alpha-hydroxylase messenger ribonucleic acid expression by luteinizing hormone and insulin-like growth factor-I. Endocrinology.

[r21] Marx TL, Mehta AE (2003). Polycystic ovary syndrome: pathogenesis and treatment over the short and long term. Cleve Clin J Med.

[r22] Matthews DR, Hosker JP, Rudenski AS, Naylor BA, Treacher DF, Turner RC (1985). Homeostasis model assessment: insulin resistance and beta-cell function from fasting plasma glucose and insulin concentrations in man. Diabetologia.

[r23] Medigović I, Ristić N, Trifunović S Manojlović-Stojanoski M, Milošević V, Zikić D, Nestorović N (2012). Genistein affects ovarian folliculogenesis: a stereological study. Microsc Res Tech.

[r24] Moutsatsou P (2007). The spectrum of phytoestrogens in nature: our knowledge is expanding. Hormones.

[r25] Myllymäki S, Haavisto T, Vainio M, Toppari J, Paranko J (2005). In vitro effects of diethylstilbestrol, genistein, 4-tert-butylphenol, and 4-tert-octylphenol on steroidogenic activity of isolated immature rat ovarian follicles. Toxicol Appl Pharmacol.

[r26] Nynca A, Sadowska A, Orlowska K, Jablonska M, Ciereszko RE (2015). The effects of phytoestrogen genistein on steroidogenesis and estrogen receptor expression in porcine granulosa cells of large follicles.. Folia Biol (Krakow).

[r27] Ohno S, Nakajima Y, Inoue K, Nakazawa H, Nakajin S (2003). Genistein administration decreases serum corticosterone and testosterone levels in rats. Life Sci.

[r28] Paixão L, Ramos RB, Lavarda A, Morsh DM, Spritzer PM (2017). Animal models of hyperandrogenism and ovarian morphology changes as features of polycystic ovary syndrome: a systematic review. Reprod Biol Endocrinol.

[r29] Pasquali R, Casimirri F, De Iasio R, Mesini P, Boschi S, Chierici R, Flamia R, Biscotti M, Vicennati V (1995). Insulin regulates testosterone and sex hormone-binding globulin concentrations in adult normal weight and obese men. J Clin Endocrinol Metab.

[r30] Pasquali R, Zanotti L, Fanelli F, Mezzullo M, Fazzini A, Morselli Labate AM, Repaci A, Ribichini D, Gambineri A (2016). Defining Hyperandrogenism in Women With Polycystic Ovary Syndrome: A Challenging Perspective. J Clin Endocrinol Metab.

[r31] Pelletier G, El-Alfy M (2000). Immunocytochemical localization of estrogen receptors alpha and beta in the human reproductive organs. J Clin Endocrinol Metab.

[r32] Rajaei S, Alihemmati Ph DA, Abedelahi Ph DA (2019). Antioxidant effect of genistein on ovarian tissue morphology, oxidant and antioxidant activity in rats with induced polycystic ovary syndrome. Int J Reprod Biomed.

[r33] Rajan RK, M SS, Balaji B (2017). Soy isoflavones exert beneficial effects on letrozole-induced rat polycystic ovary syndrome (PCOS) model through anti-androgenic mechanism. Pharm Biol.

[r34] Ren MQ, Kuhn G, Wegner J, Nakagomi M, Usumi K, Ono H (2001). Isoflavones, substances with multi-biological and clinical properties. Eur J Nutr.

[r35] Sanfilippo S, Canis M, Ouchchane L, Botchorishvili R, Artonne C, Janny L, Brugnon F (2011). Viability assessment of fresh and frozen/thawed isolated human follicles: reliability of two methods (Trypan blue and Calcein AM/ethidium homodimer-1). J Assist Reprod Genet.

[r36] Schoeters G, Den Hond E, Dhooge W, van Larebeke N, Leijs M (2008). Endocrine disruptors and abnormalities of pubertal development. Basic Clin Pharmacol Toxicol.

[r37] Setchell KD (2006). Assessing risks and benefits of genistein and soy. Environ Health Perspect.

[r38] Shi D, Vine DF (2012). Animal models of polycystic ovary syndrome: a focused review of rodent models in relationship to clinical phenotypes and cardiometabolic risk. Fertil Steril.

[r39] Skrtic A, Sokolic L, Borovecki A Rosa J, Fenzl V (2011). Immunohistochemical localization of CD31, NOTCH1 and JAGGED1 proteins in experimentally induced polycystic ovaries of immature rats. Acta Histochem.

[r40] Sun J, Jin C, Wu H, Zhao J, Cui Y, Liu H, Wu L, Shi Y, Zhu B (2013). Effects of electro-acupuncture on ovarian P450arom, P450c17α and mRNA expression induced by letrozole in PCOS rats. PLoS One.

[r41] Tena G, Moran C, Romero R, Moran S (2011). Ovarian morphology and endocrine function in polycystic ovary syndrome. Arch Gynecol Obstet.

[r42] Theil C, Briese V, Gerber B, Richter DU (2011). The effects of different lignans and isoflavones, tested as aglycones and glycosides, on hormone receptor-positive and -negative breast carcinoma cells in vitro. Arch Gynecol Obstet.

[r43] Tosi F, Negri C, Perrone F, Dorizzi R, Castello R, Bonora E, Moghetti P (2012). Hyperinsulinemia amplifies GnRH agonist stimulated ovarian steroid secretion in women with polycystic ovary syndrome. J Clin Endocrinol Metab.

[r44] Weber KS, Setchell KD, Stocco DM, Lephart ED (2001). Dietary soy-phytoestrogens decrease testosterone levels and prostate weight without altering LH, prostate 5alpha-reductase or testicular steroidogenic acute regulatory peptide levels in adult male Sprague-Dawley rats. J Endocrinol.

[r45] Zhuang XL, Fu YC, Xu JJ, Kong XX, Chen ZG, Luo LL (2010). Effects of genistein on ovarian follicular development and ovarian life span in rats. Fitoterapia.

[r46] Zin SR, Omar SZ, Khan NL, Musameh NI, Das S, Kassim NM (2013). Effects of the phytoestrogen genistein on the development of the reproductive system of Sprague Dawley rats. Clinics.

